# Characterization of Powdered Roasted Açaí Seeds (*Euterpe oleracea* Mart.): Impact on Bioactive Compounds and Nutritional Potential

**DOI:** 10.1002/fsn3.72353

**Published:** 2026-09-16

**Authors:** Renata da Silva Magalhães, Gabriel Sthefano Lourenço Pereira, Sara Fraga, Patrícia Tonon de Souza, Dhayna Oliveira Sobral, Marcelo Antônio Morgano, Antonio José de Almeida Meirelles, Guilherme José Maximo, Eduardo Augusto Caldas Batista, Klicia Araujo Sampaio

**Affiliations:** ^1^ Laboratory of Extraction, Applied Thermodynamics and Equilibrium (EXTRAE), Department of Food Engineering and Technology, School of Food Engineering Universidade Estadual de Campinas (UNICAMP) Campinas São Paulo Brazil; ^2^ Food Science and Quality Center (CCQA) Institute of Food Technology (ITAL) Campinas São Paulo Brazil

**Keywords:** antioxidants, aqueous extract, oil extract, proximate composition, roasting

## Abstract

This study evaluated the effects of roasting on the physical, chemical, and bioactive properties of açaí seeds ( *Euterpe oleracea* Mart.). Roasted seed powder presented an average particle size of 432.55 μm, real density of 1.51 g/cm^3^, and porosity of 0.43, along with low lipid content (1.8 g/100 g) and high dietary fiber (48 g/100 g). Relevant mineral levels were also observed, particularly calcium (67 mg/100 g), potassium (443 mg/100 g), and phosphorus (152 mg/100 g). The lipid fraction was obtained by Soxhlet extraction, while an aqueous extract was produced via optimized solid–liquid extraction, yielding two fractions for comparative analysis. High phenolic contents were detected in both, the oil (1387.13 ± 17.83 mg CAE/g) and aqueous extract (1272.3 ± 3.26 mg CAE/g), with significant antioxidant activity measured by DPPH (IC_50_: 257.82 ± 0.91 and 227.20 ± 3.69 mg/mL, respectively) and FRAP assays (1344.59 ± 17.67 and 2365.07 ± 2.46 μmol FeSO_4_/100 g, respectively). The lipid fraction contained α‐tocopherol and tocotrienols (α‐, γ‐, and δ‐), indicating its potential as a source of vitamin E‐active compounds, and was mainly composed of oleic, linoleic, myristic, and palmitic acids. Thermal analysis revealed multiple crystallization events and a melting point near 30°C, suggesting a complex triacylglycerol profile. These findings highlight roasted açaí seeds as a promising agro‐industrial by‐product with relevant nutritional and functional properties, supporting their potential application in food and related industries.

## Graphical abstract

Roasting enhanced the functional potential of açaí ( *Euterpe oleracea* Mart.) seeds, yielding a fiber‐rich powder with relevant minerals and bioactive compounds. Both lipid and aqueous extracts showed high phenolic contents and antioxidant activity, while the lipid fraction provided vitamin E‐active compounds and valuable fatty acids. These findings highlight roasted açaí seeds as a promising agro‐industrial by‐product for developing functional food ingredients and other value‐added applications.

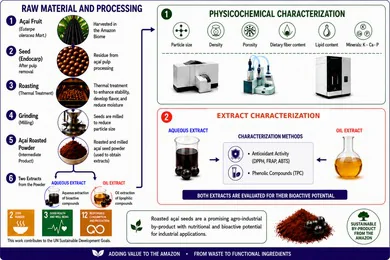

## 1 Introduction

In recent years, açaí has attracted increasing global attention due to its high antioxidant capacity and rich bioactive composition. Derived from the palms *Euterpe oleracea* and *Euterpe precatoria*, native to the Amazon region, the fruit is characterized by its dark purple color and a large seed measuring approximately 2 cm in diameter. Açaí pulp finds broad application across the food industry, from juices and frozen desserts to jellies and functional beverages, as well as in pharmaceutical and cosmetic formulations (Gordon et al. 2012).

Brazil is the world's leading producer of açaí, with the states of Pará, Amazonas, and Amapá accounting for most of the national production (IBGE 2024). The growing commercial demand for açaí has resulted in the generation of large amounts of agro‐industrial residue, since the seed represents approximately 80% of the fruit's total weight (Pompeu et al. 2009; Neves and Camargo 2015). Despite their by‐product status, açaí seeds hold potential for application in animal feed, organic fertilizers, biorefineries, functional food development, and bioactive compound recovery (Alves et al. 2022; De Oliveira et al. 2012; Lima 2015).

Although extensive research has explored the nutritional and functional properties of raw açaí seeds, knowledge about the effects of roasting on their composition and antioxidant‐related properties remains limited. Roasting is a thermal process known to induce complex physicochemical changes in plant matrices. Maillard reactions between reducing sugars and amino acids can generate new antioxidant compounds, whereas degradation of heat‐labile phenolics may reduce certain fractions (Farah and Donangelo 2006). Roasting may also disrupt cell wall structures and protein–phenolic complexes, potentially releasing previously bound bioactive compounds and altering their extractability and bioaccessibility (Castaldo et al. 2012).

Despite the commercial availability of açaí seed powder as a caffeine‐free coffee substitute, no study has yet systematically evaluated how roasting transforms the nutritional composition as well as the phenolic profile, tocol composition, fatty acid stability, and thermal behavior of extracts obtained by them. Addressing this gap is therefore essential not only for advancing the scientific understanding of açaí seed chemistry but also for enabling its informed use as a functional food ingredient. To address this, the present study provides a comprehensive physicochemical and nutritional characterization of powdered roasted açaí seeds, as well as their aqueous and oil extracts, including evaluation of their mineral profile, total phenolic content and antioxidant activity, tocopherols, tocotrienols, fatty acid and acylglycerols profile, and thermal behavior. These findings contribute to elucidating the nutritional and functional potential of roasted açaí seeds, supporting their valorization as a functional food ingredient.

## 2 Material and Methods

### 2.1 Sample Preparation

**FIGURE 1:**
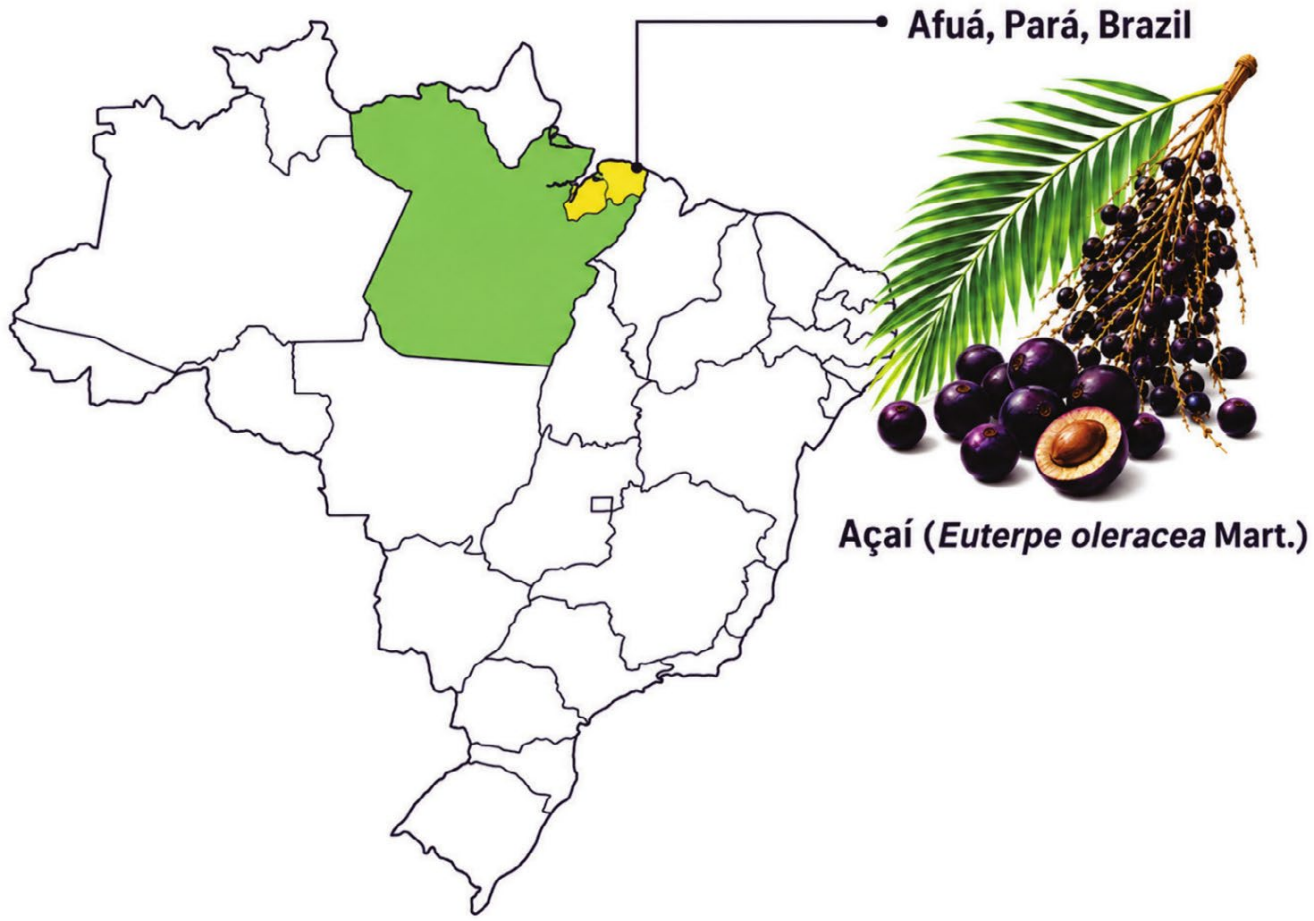
Geographic location of Afuá (Marajó Archipelago, Pará, Brazil) within the Amazon biome, highlighting the origin of the *Euterpe oleracea* Mart. (açaí) seeds used in the study.

Roasted açaí seeds were supplied by the company Raízes do Açaí, located in Afuá, Pará, Brazil (00°′09′S, 50°23′W; altitude ~5 m), where the seeds were roasted at 140°C for 60 min, following the company's standard commercial production protocol. The geographic origin of the raw material is illustrated in Figure 1. Afuá is located in the Marajó Archipelago, an equatorial region classified as Af (tropical rainforest climate) according to the Köppen classification, characterized by mean annual temperatures ranging from 24°C to 32°C, relative humidity between 80% and 90%, and annual precipitation between 2000 and 3000 mm (Alvares et al. 2013).

**FIGURE 2:**
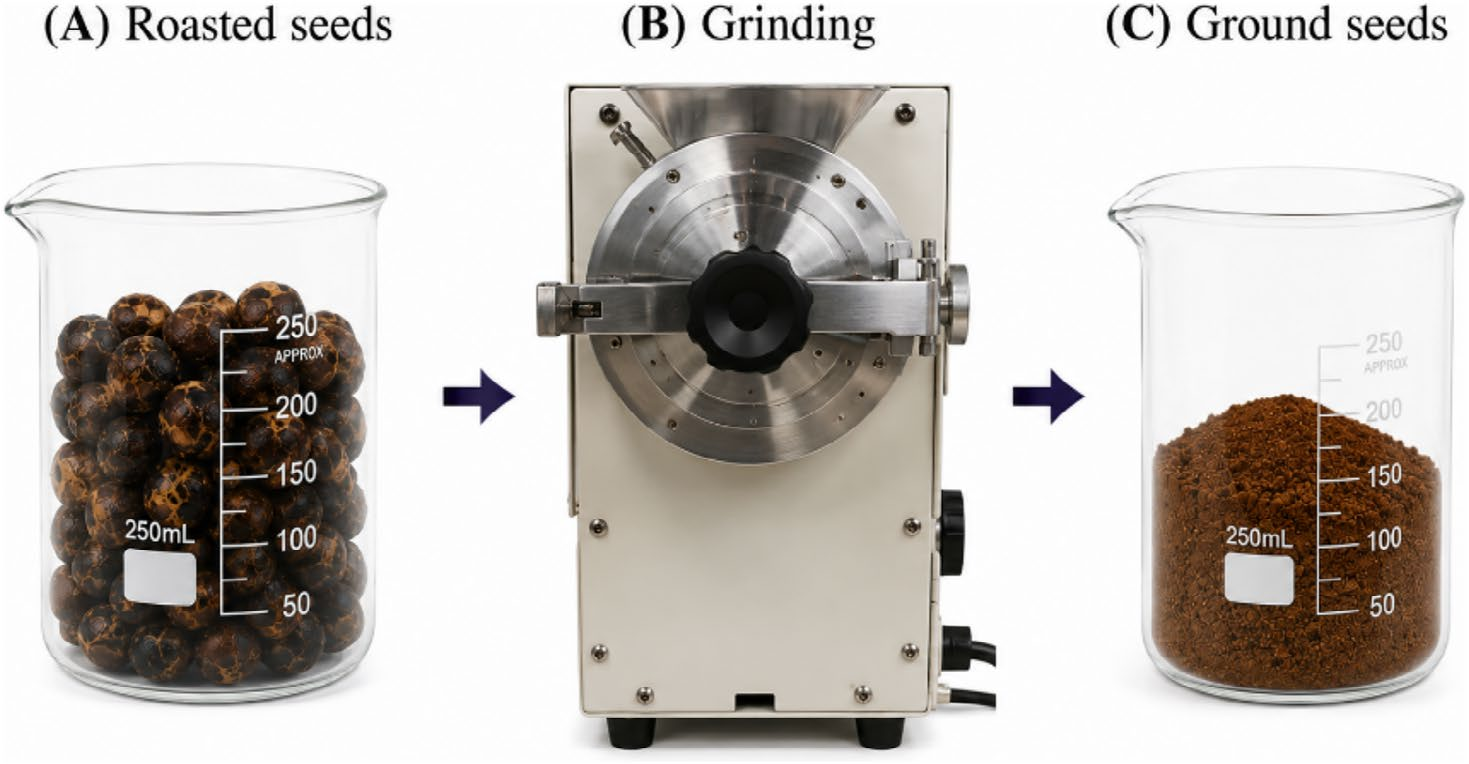
Sample preparation process of roasted açaí seeds: (A) Roasted seeds, (B) grinding, (c) ground seeds.

Upon arrival, the roasted seeds (Figure 2) were ground using a knife mill (Marconi MA340, Brazil), packaged in light‐protected bags, and stored at 4.5°C until analysis. All analyses were conducted at the Laboratory of Extraction, Applied Thermodynamics and Equilibrium (ExTrAE), Universidade Estadual de Campinas (UNICAMP), Campinas, São Paulo, Brazil.

### 2.2 Physicochemical Characterization of Roasted Açaí Seed Powder

The volumetric mean diameter of the roasted açaí seed powder was determined using a Mastersizer particle size analyzer (Malvern, model Mastersizer 2000, United Kingdom). The real particle density $\left(\rho_{r}\right)$ was measured using a helium gas pycnometer (Micromeritics, model AccuPyc II 1340, USA), and the apparent bed density of the particles $\left(\rho_{a}\right)$ was calculated using the cup method (Uquiche et al. 2004). The bed porosity $\left(\varepsilon \right)$ was calculated according to the equation: $\varepsilon =1-\left(P_{a}/P_{r}\right).$

The moisture content was determined by the AOCS Ca 2e‐84 method (AOCS 2017) using a KF Titrino Plus apparatus (model 870, Metrohm, Switzerland) equipped with a Thermoprep KF furnace (model 832, Metrohm, Switzerland). The total moisture and volatile compounds content was measured by the gravimetric method 930.04 of AOAC (2012). The ash content was obtained by sample calcination in a furnace at 550°C, followed by weighing the resulting residue (AOCS Ba 5a‐49, 2017). The lipid content was extracted using an Ankom extractor (ANKOM Technology, model XT15, USA) according to the AOCS Am 5–04 method (2017). Total proteins were determined by the DUMAS combustion method (model NDA 701, VELP, United Kingdom). The carbohydrate content was calculated by difference following RDC n°. 360/2003 (Brasil 2003), and crude fiber was determined by acid and alkaline digestion of the dried and defatted sample (AOCS Ba 6a‐05, 2017). Atwater coefficients were used to determine the caloric value (Merrill and Watt 1973), and pH was measured potentiometrically with a digital benchtop pH meter (Metrohm, model 913, Switzerland).

For the determination of calcium, copper, iron, phosphorus, magnesium, manganese, potassium, sodium, and zinc, the following procedure was performed: 2.5 g of the sample was weighed and heated on a heating plate, incinerated for 10 h at 450°C. The resulting ashes were moistened with purified reverse osmosis water and mixed with hydrochloric acid to quantitatively transfer them to a 25 mL volumetric flask. An analytical blank was prepared in the same manner, but without adding the sample, and tests were conducted in triplicate following AOAC 985.35 and 984.27 standards from 2012.

### 2.3 Production of Extracts

#### 2.3.1 Aqueous Extract

The aqueous extract of roasted açaí seeds was obtained by solid–liquid extraction using ultrapure water (Milli‐Q, Germany) and a filter paper with a weight of 80 g m^−2^. The process was conducted using the optimal extraction conditions previously established by Da Silva et al. (2024). Briefly, approximately 10 g of roasted açaí seed powder was extracted at the optimized temperature of 94.1°C with an optimized solid‐to‐solvent ratio of 10:135.25 (g mL^−1^).

#### 2.3.2 Oil Extract

The oil extract of the roasted açaí seed powder was obtained using the Soxhlet method, as detailed by Aguiar et al. (2014). Hexane with 95% purity (ACS, Brazil) was used as the solvent at atmospheric pressure. Approximately 10 g of the sample was packed into filter paper and placed in the Soxhlet extractor containing 150 mL of hexane preheated to 50°C. The extraction was carried out at a controlled temperature of 50°C using a thermostatic water bath (Paar Physica, model Viscotherm VT 2, Germany) set at 5°C, and the reflux was maintained for 5 h. After extraction, the separation of oil and solvent was achieved using a rotary evaporator (Marconi, MA‐120, Brazil) at 60°C ± 2°C under controlled vacuum pressure.

### 2.4 Characterization of the Extracts

#### 2.4.1 Total Phenolic Content

The total phenolic content (TPC) was determined in both oil and the aqueous extracts. For the oil samples, a caffeic acid stock solution (1 mg/mL) and a 10% (w/v) Na_2_CO_3_ solution were prepared. Analytical curves were constructed by diluting caffeic acid at different concentrations, and the absorbance was measured at 725 nm after reaction with the Folin–Ciocalteu reagent and Na_2_CO_3_. Oil samples (3 to 5 g) were extracted with methanol, and the absorbance of the methanolic phase was measured at 725 nm using a UV–VIS spectrophotometer (Thermo Scientific Orion, model AquaMate 8000 UV–VIS) against the established standard curve.

For the aqueous extracts, the total phenol content was determined according to Singleton et al. (1999), using the Folin–Ciocalteu reagent in an alkaline medium. Calibration curves were prepared by adding different volumes of gallic acid standard solution to volumetric flasks, with Folin–Ciocalteu reagent, sodium carbonate solution, and distilled water. After shaking and resting for 30 min, readings were performed at 760 nm in the spectrophotometer.

The results were expressed in milligrams of Caffeic Acid Equivalents (CAE) per gram of extract and oil (mg CAE/g), based on the studies by Szydłowska‐Czerniak et al. (2008) and Haiyan et al. (2007).

#### 2.4.2 Antioxidant Activity by DPPH and FRAP

The antioxidant activity was determined for both oil and the aqueous extracts. For oil extracts, the radical‐scavenging capacity (DPPH) was assessed based on the methods described by Szydłowska‐Czerniak et al. (2008); Espín et al. (2000); and Valavanidis et al. (2004), with modifications. Using DPPH solutions prepared in methanol and ethyl acetate, three distinct extract phases were obtained: a methanolic phase, recovered by centrifugation of an oil–methanol mixture, and lipidic and total phases, both isolated via ethyl acetate partitioning. Each phase was tested at varying concentrations in Falcon tubes, and the degree of radical inhibition was determined spectrophotometrically at 515 nm following a 30 min reaction period. Antioxidant capacity was reported as IC_50_ (mg/mL), representing the sample concentration needed to inhibit 50% of the DPPH radical activity. For the aqueous extract, the DPPH method of Brand‐Williams et al. (1995) was applied, with absorbance readings taken at 517 nm. Dose–response inhibition curves were plotted and IC_50_ values derived accordingly.

Electron‐transfer antioxidant capacity was further characterized for both extracts through the ferric reducing antioxidant power (FRAP) assay, performed on the same methanolic extracts. Briefly, 0.3 mL of each extract was combined with 2.0 mL of FRAP reagent and 7.7 mL of distilled water, and the resulting mixture was incubated at 25°C for 10 min in the absence of light. Samples were then centrifuged at 10.000 rpm for 10 min, and absorbance was recorded at 593 nm against a blank consisting of 2.0 mL of FRAP reagent and 8.0 mL of distilled water. Quantification was based on a calibration curve prepared with FeSO_4_·7H_2_O in distilled water (0.00025–0.08 mmol/L), a solvent chosen due to the poor solubility of the FRAP reagent in methanol and acetone. Results were expressed as μmol FeSO_4_ equivalents per 100 g of sample (Espín et al. 2000).

#### 2.4.3 Fatty Acid Profile

The fatty acid profile of the oil extracts was determined by gas chromatography, according to the AOCS Ce 1–62 method (2017). The equipment used was an Agilent gas chromatograph (model 7890B, USA) with a flame ionization detector (FID) and an Agilent capillary column (model DB‐WAX, USA), with the following specifications: 30 m in length, 0.25 mm in internal diameter, and 0.25 μm in film thickness. The carrier gas used was helium, with a flow rate of 25 mL/min. The column temperature was programmed as follows: initial ramp from 50°C to 120°C at 15°C/min, held for 15 min; then 4°C/min to 200°C, held for 3 min; and finally 4°C/min to 250°C. The injector temperature was 250°C, and the detector temperature was 300°C. The injection volume was 1 μL. The conversion of fatty acids into methyl esters (FAME) was performed according to the methodology of Metcalfe et al. (1966), adapted by Hartman and Lago (1973). Peak identification was done by comparison with the retention times of FAME standards (C8–C24; Sigma Aldrich, USA).

#### 2.4.4 Mono‐, Di‐, and Triacylglycerols Content

Mono (MAG) and diacylglycerols (DAG) content for oil extracts were determined by gas chromatography, following the method described in AOCS Cd 11‐b‐91 (2017). Using the same gas chromatograph mentioned above, helium was used as the carrier gas at a flow rate of 5 mL/min, and the column temperature was programmed to vary from 60°C to 360°C. MAG and DAG contents were calculated based on calibration curve equations obtained by summing the areas of the retention times. The content of triacylglycerol (TAG) was determined by the difference between 100 and the sum of the MAG and DAG contents.

The composition of triacylglycerols (TAG) was determined from fatty acid (FA) data through combinatorial analysis, as described Antoniosi Filho et al. (1995). The trisaturated TAG content, an information demanded in the method, was estimated at 1.13%, based on the value reported by Silva et al. (2017) for jussara oil (*Euterpe edulis* Mart.), a palm of the same genus as açaí.

#### 2.4.5 Melting and Crystallization Profile

The thermal behavior of the oil extracts was analyzed by differential scanning calorimetry (DSC) using the DSC 2920 calorimeter (TA Instruments, New Castle, USA), coupled with a cooling system using nitrogen as the purge gas, according to a method adapted from AOCS Cj 1–94 (AOCS 2017). Samples, weighing between 3 and 5 mg, were placed in aluminum pans, with an empty and hermetically sealed pan used as reference. The analysis conditions included maintaining the samples at 70°C for 10 min, followed by cooling at a rate of 5°C/min to −60°C, maintaining that temperature for 30 min (Tan and Che Man 2002). After this cycle, the samples were subjected to a melting process from −60°C to 70°C at a rate of 5°C/min.

#### 2.4.6 Tocopherol and Tocotrienol Profile

The analysis of tocols (tocopherols and tocotrienols) of the oil extracts was carried out using ultra high performance liquid chromatography electrospray ionization—mass spectrometry (UHPLC/ESI/MS) on a Waters Acquity system (USA), following the protocol described by Ansolin et al. (2017). For the UHPLC analysis, a C18 column (model UPLC BEH C18, Acquity, USA) was utilized, employing a mobile phase composed of methanol: water: ammonium hydroxide (99:1:0.1 v/v/v) and isopropanol. Prior to analysis, samples of açaí oil were diluted in isopropanol of HPLC grade. Instrument control and data acquisition were conducted using MassLynx software. The obtained correlation coefficients exceeded 0.99, indicating the method's satisfactory linearity.

### 2.5 Statistical Analysis

All analyses were performed in triplicate (*n* = 3) and results are expressed as mean ± standard deviation (SD). Since this study characterizes a single commercially produced sample with no independent treatment groups, descriptive statistics (mean, SD, and RSD%) were applied to proximate composition and mineral data (Granato et al. 2014). Differences among tocol compounds were assessed by one‐way ANOVA with Tukey's test (Statistica 14.0; StatSoft Inc. 2023). TAG compositions were determined by combinatorial analysis in MATLAB (Version 6.0, MathWorks).

## 3 Results and Discussion

### 3.1 Characterization of the Roasted Açaí Seed Powder

The particle size analysis of roasted açaí seed powder revealed an average size of 432.55 μm, as shown in Table 1. In comparison, the particle size of coffee powder can range from 80 to 840 μm, depending on the grinding method and consumer preferences for beverage preparation. This characteristic is essential for assessing the quality of roasted seed powder, as particle size directly influences the extraction, oxidation, and degradation of bioactive compounds. Larger particles tend to reduce extraction and to protect the powder from oxidation due to a smaller surface area exposed to oxygen. Conversely, smaller particles present a larger exposed surface area that can enhance extraction but can also accelerate the degradation of sensitive compounds.

**TABLE 1:** Physical properties, proximate composition (g/100 g), and mineral profile (mg/100 g) of roasted açaí seed powder.

Parameter	Mean ± SD	RSD%
*Physical properties*		
Particle size (μm)	432.55 ± 0.24	0.06
Real density (g/cm^3^)	1.51 ± 0.00	0.33
Bulk density (g/cm^3^)	0.86 ± 0.00	0.58
Porosity	0.43	—
*Proximate composition (g/100 g)*		
Lipids^a^	1.8 ± 0.19	10.56
Carbohydrates	38.0 ± 0.13	0.34
Crude fiber (d.b.)	48.0 ± 0.45	0.94
Proteins	5.2 ± 0.12	2.31
Ash	1.65 ± 0.13	7.88
Moisture	4.3 ± 0.07	1.63
Caloric value (kcal/100 g)	380.85 ± 0.32	0.08
pH	5.0 ± 0.00	0.10
*Minerals (mg/100 g)*		
Calcium	67.0 ± 1.0	1.49
Copper^a^	1.77 ± 0.19	10.73
Iron	1.5 ± 0.03	2.00
Phosphorus	152.0 ± 2.0	1.32
Magnesium	16.0 ± 1.0	6.25
Manganese	16.0 ± 1.0	6.25
Potassium	443.0 ± 6.0	1.35
Sodium	4.27 ± 0.36	8.43
Zinc	1.28 ± 0.02	1.56

Abbreviations: —, not applicable; d.b., dry basis.

^a^ RSD% > 10%: lipids (Soxhlet extraction variability) and copper (low absolute concentration in the matrix); both values remain within the acceptable threshold of ≤ 15% (AOAC 2012).

Although no studies in the literature specifically address roasted açaí seed powder, the roasting, grinding, and preparation processes follow principles analogous to those used for coffee. Therefore, the comparison with coffee is relevant, particularly when considering the effects of particle size on product quality. Roasting and grinding also affect the microstructure and extraction parameters of compounds, characteristics that apply to both coffee and roasted açaí seeds. However, it is important to emphasize that this study investigates a novel product, the “coffee” from roasted açaí seeds, filling a gap in the scientific literature. Therefore, in addition to comparisons with coffee, further investigations are required to explore the specific properties of roasted açaí seeds to elucidate the particularities of this new Amazonian product.

Density is a key indicator in food processing, packaging, storage, and transportation, as it reflects the amount of material by weight in each volume. Although the study by Nakilcioğlu‐Taş and Ötleş (2019) investigated the particle density of coffee powder, it is pertinent to establish a comparison with roasted açaí seed powder, as both products undergo similar roasting and grinding processes. In the case of coffee powder, the particle density ranges from 0.91 to 0.92 g/cm^3^, with a variation of approximately ±0.012 g/cm^3^ for dark roasted coffee and ± 0.015 g/cm^3^ for raw coffee. Compacted density also showed variations, ranging from 0.34 ± 0.007 g/cm^3^ for dark roasted coffee to 0.52 ± 0.019 g/cm^3^ for raw coffee. These variations are directly related to roasting processes, which affect the internal structure of the particles and, consequently, their density.

The porosity result for roasted açaí seed powder was 0.43, a value calculated by dividing the void volume by the total volume. Porosity is an important physical property in agro‐industrial processes related to solvent flow in solid–liquid extraction and the resistance of a product layer to air movement and is often used in the design of equipment for drying and storing grains, seeds, and powders.

As shown in Table 1, the powder was characterized by low lipid content (1.8 ± 0.19 g/100 g) and a slightly acidic pH (5.0). The main constituents identified were carbohydrates (38 ± 0.13 g/100 g) and fibers (48 ± 0.45 g/100 g). In the study by Da Silva et al. (2024), the same roasted powder was also employed to evaluate the presence of inulin in aqueous extracts, confirming it as a prebiotic source. Response surface analysis defined the optimal extraction conditions as 94.1°C and a solid‐to‐solvent ratio of 10:135.25 g·mL^−1^, resulting in an experimental inulin yield of 166.38 mg·g^−1^, a value very close to the model prediction (164.57 mg·g^−1^; relative deviation of 0.57%), thereby validating the method. The high fiber and inulin contents highlight the potential of roasted açaí seeds, commonly discarded as waste in urban and agro‐industrial markets in northern Brazil, as a sustainable source of polysaccharides for industrial applications and as a functional ingredient for enriching foods with dietary fiber.

The current study found higher values for carbohydrates (+0.29 g/100 g), proteins (+0.09 g/100 g), lipids (+0.06 g/100 g), ash (+0.02 g/100 g), and fibers (+1.10 g/100 g) compared to Da Costa et al. (2020). These variations in the proximate composition of açaí seed powder may be attributed to differences in soil and climatic conditions in the regions where the açaí fruits for this study were harvested compared to those in previous studies. Conversely, the data demonstrated greater alignment with the results obtained by Alves et al. (2022), who analyzed raw açaí seed flour. Their study reported ash content (1.64 ± 0.29 g/100 g), lipids (2.8 ± 0.58 g/100 g), moisture (5.53 ± 0.28 g/100 g), proteins (5.32 ± 0.32 g/100 g), carbohydrates (90.24 ± 0.5 g/100 g), caloric value (407.43 ± 3.63 kcal/100 g), fibers (84.14 ± 0.21 g/100 g), and pH (5.82 ± 0.02), which are consistent with the findings of other studies, such as those by Melo et al. (2021) and Domingues et al. (2017).

The variations in the proximate composition of açaí seeds reported in different studies may reflect differences between species, environmental conditions, and post‐harvest processing. In the present study, roasted seeds of wild *Euterpe oleracea* were obtained in Afuá (Marajó Island, Pará), a floodplain ecosystem characterized by permanent fluvial influence and highly suitable environmental conditions for this species. In contrast, Alves et al. (2022) evaluated *Euterpe precatoria*, a distinct species naturally adapted to Western Amazonia and the transition to the Cerrado, where differences in soil composition, rainfall regime, and temperature may influence carbohydrate deposition and mineral accumulation in the palm seeds. Although Melo et al. (2021) analyzed samples in southeastern Brazil, the raw material likely originated from commercial supply chains based in Pará, the main producing region of *E. oleracea* in Brazil. Therefore, the compositional differences between the studies may not exclusively reflect phytogeographical variation, but also differences in processing conditions, roasting parameters, and sample handling. Together, these factors may explain the observed variations in fiber, lipid, and mineral content among the studies. Future investigations should systematically compare roasted açaí seeds from different species and well‐defined geographic origins to establish a reliable compositional database for this agro‐industrial byproduct.

The mineral composition of roasted açaí seed powder is noteworthy from a dietary standpoint (Table 1). Calcium (67 ± 1 mg/100 g), potassium (443 ± 6 mg/100 g), phosphorus (152 ± 2 mg/100 g), iron (1.5 ± 0.03 mg/100 g), magnesium (16 ± 1 mg/100 g), manganese (16 ± 1 mg/100 g), and zinc (1.28 ± 0.02 mg/100 g) were all detected at concentrations that fall within ranges considered nutritionally relevant for a balanced diet, particularly when this powder is consumed regularly as a coffee‐like beverage.

However, the values for copper (1.77 ± 0.19 mg/100 g) and sodium (4.27 ± 0.36 mg/100 g) deserve special attention. The Tolerable Upper Intake Level (UL) for copper established by the Institute of Medicine is 10 mg/day for adults (Institute of Medicine (US) Panel on Micronutrients 2001). Considering a typical serving of 10 g of powder per beverage preparation (analogous to coffee), the copper contribution per serving would be approximately 0.177 mg, representing ~20% of the Estimated Average Requirement (EAR = 0.7 mg/day) and well below the UL. However, individuals with hepatic conditions or Wilson's disease, a genetic disorder impairing copper metabolism, should exercise caution (Ala et al. 2007). Regarding sodium, although the absolute content is low in comparison to processed foods (dietary reference intake for sodium is 2300 mg/day for adults; U.S. Department of Agriculture and U.S. Department of Health and Human Services 2020), regular and high‐volume consumption could contribute incrementally to daily intake, particularly in individuals following sodium‐restricted diets. Therefore, while moderate consumption of açaí seed powder is considered safe for the general population, a recommended serving size and frequency of use should be further investigated in future studies.

### 3.2 Evaluation of Extracts

#### 3.2.1 Total Phenolic Content and Antioxidant Activity

Roasting had a marked effect on the phenolic content of both the aqueous and oil extracts, with values reaching 1272.3 ± 3.26 mg CAE/g and 1387.13 ± 17.83 mg CAE/g, respectively. These concentrations point to a substantial mobilization of phenolic compounds during thermal processing, a phenomenon well described for other roasted plant matrices. At temperatures above 100°C, Maillard reactions between reducing sugars and amino acids produce melanoidins, brown pigments whose radical‐scavenging activity has been repeatedly documented (Farah and Donangelo 2006). Beyond this, heat‐driven hydrolysis of ester and glycosidic linkages can release phenolic acids that were previously bound within the cell wall, making them extractable for the first time (Naczk and Shahidi 2004). Physical disruption of the seed's compact fibrous matrix by roasting increases bed porosity, which in turn facilitates solvent access to otherwise inaccessible phenolic pools. This last mechanism may be especially relevant in açaí seeds, which are naturally rich in condensed tannins tightly associated with insoluble fiber (Melo et al. 2021). Comparable effects have been reported for coffee by‐products: Guglielmetti et al. (2019) recovered 81.03 and 19.67 mg CAE/g from silver skin and husk through aqueous extraction at 100°C, while Castaldo et al. (2012) obtained 31.2 mg/g from silver skin under similar conditions. That said, uncontrolled or excessive heat can degrade heat‐labile phenolics, which underscores the relevance of standardized roasting protocols.

Radical‐scavenging capacity, assessed by the DPPH assay, revealed that the aqueous extract exhibited moderately greater activity than the oil fraction, with IC_50_ values of 227.20 ± 3.69 mg/mL and 257.82 ± 0.91 mg/mL, respectively. This pattern is consistent with the higher total phenolic concentration observed in the aqueous extract and its greater abundance of water‐soluble radical‐scavenging compounds. When compared with raw açaí seed matrices, the IC_50_ values obtained in the present study are numerically higher than those reported by Melo et al. (2021) (105.91 mg/mL) and Martinez et al. (2018) (15.66 ± 0.44 mg/mL). These differences most likely reflect methodological disparities in extraction conditions, including temperature, solid‐to‐solvent ratio, and solvent polarity, as well as roasting‐induced compositional shifts in the phenolic profile rather than an inherent reduction in antioxidant capacity.

The Ferric Reducing Antioxidant Power (FRAP) assay was employed to complement the radical‐scavenging data with an assessment of electron‐transfer capacity. The aqueous extract demonstrated a FRAP value of 2365.07 ± 2.46 μmol FeSO_4_/100 g, while the oil fraction exhibited a robust reducing capacity of 1344.59 ± 17.67 μmol FeSO_4_/100 g, values that substantially exceeded those reported for standard commercial reference oils (64.07 μmol FeSO_4_/100 g) and conventional hexane extracts obtained by Soxhlet (238.78 μmol FeSO_4_/100 g).

These results are considerably higher than the only FRAP value currently available in the literature for roasted açaí seed extract (183.33 μmol TE/g; Lima et al. 2025), a discrepancy attributable to differences in extraction conditions, solid‐to‐solvent ratios, and calibration standards: the present study used FeSO_4_·7H_2_O as a reference standard, while the cited work expressed the results in Trolox equivalents, which prevents direct numerical comparison. A similar interpretive caveat applies to comparisons of total phenolic content: Lima et al. (2025) reported a TPC of 21.78 mg GAE/g using gallic acid as a calibration standard, while the present study expressed the results in mg CAE/g using caffeic acid. Since gallic acid and caffeic acid have distinct molar absorptivities in the Folin–Ciocalteu assay, these values are not directly interconvertible, and therefore the apparent numerical differences between studies should be interpreted with caution. For raw seed matrices, Martinez et al. (2018) also reported elevated FRAP activity, along with a total phenolic content of 37.08 ± 8.56 g gallic acid/100 g, suggesting that ferric‐reducing capacity is an intrinsic characteristic of açaí seed phenolics that may be further amplified by roasting. Taken together, the convergence of DPPH and FRAP results, encompassing hydrogen‐atom transfer (HAT) and electron‐transfer (ET) mechanisms, respectively, offers a more comprehensive and mechanistically grounded characterization of the antioxidant profile of roasted açaí seed extracts and oil, consistent with the co‐occurrence of water‐soluble phenolics and lipophilic tocotrienols identified in this study (Prior et al. 2005; Apak et al. 2016).

### 3.3 Tocopherols and Tocotrienols Profile

Four tocol compounds were detected in roasted açaí seed oil extract: α‐tocopherol, α‐tocotrienol, γ‐tocotrienol, and δ‐tocotrienol, with α‐tocotrienol predominating (Table 2). This finding contrasts with reports on raw açaí seeds, where tocopherols were not detected at all (Alves et al. 2022), suggesting that roasting plays a decisive role in their extractability. Seasonal variation and geographic origin of the raw material may also contribute to the observed profile, as both factors are known to influence tocol biosynthesis in palm species. Gao et al. (2021) similarly found that roasting increased total tocopherol content in walnut oil, attributing this to the thermal release of tocols previously bound to proteins and phospholipids, a pattern that appears applicable to açaí seeds as well.

**TABLE 2:** Tocol profile of roasted açaí seed oil extract.

Compound	Concentration (μg/g)	Tukey (*p* < 0.05)
α‐tocotrienol	12.17 ± 0.16	a
γ‐tocotrienol	8.83 ± 0.17	b
α‐tocopherol	2.81 ± 0.05	c
δ‐tocotrienol	0.47 ± 0.06	d
β/γ‐tocopherol	Nd	—
δ‐tocopherol	Nd	—

*Note:* Different lowercase letters indicate significant differences among detected compounds (Tukey's test, p < 0.05). Values expressed as mean ± SD (*n* = 3).

Abbreviations: —, not included in statistical comparison; Nd, not detected.

Statistically significant differences were observed among all four detected tocol compounds (Tukey's test, *p* < 0.05; Table 2). α‐Tocotrienol was the predominant tocol (12.17 ± 0.16 μg/g; group a), followed by γ‐tocotrienol (8.83 ± 0.17 μg/g; group b), α‐tocopherol (2.81 ± 0.05 μg/g; group c), and δ‐tocotrienol (0.47 ± 0.06 μg/g; group d). The significant predominance of α‐tocotrienol over all other forms supports the discussion of roasted açaí seed oil as a promising source of vitamin E‐active compounds, particularly tocotrienols, as detailed below.

During roasting, elevated temperatures act on the seed matrix through several interconnected pathways. Protein denaturation and partial hydrolysis free tocols previously anchored to structural complexes, while disruption of phospholipid bilayers and oil body membranes increases their accessibility to organic solvents during extraction. Redistribution from more polar cellular compartments into the neutral lipid fraction further concentrates these compounds in the extractable oil phase (Gao et al. 2021; Wroniak et al. 2016). Furthermore, the unsaturated triene side chain of tocotrienols confers greater membrane mobility compared to tocopherols, favoring a more homogeneous distribution within lipid bilayers and potentially enhancing radical‐scavenging efficiency in membrane systems (Londoño et al. 2025). The apparent thermal stability of these compounds at 140°C, together with their increased extractability after roasting, highlights roasted açaí seed oil as a promising source of vitamin E‐active compounds.

The EFSA ANS Panel (EFSA Panel on Food Additives and Nutrient Sources added to Food) (2015) noted that γ‐tocopherol is the predominant isomer in seeds and oils, representing more than 70%, while α‐tocopherol is found in a greater proportion in leaf tissues, at more than 90%. Both isomers are active as vitamin E. Tocopherols are lipophilic antioxidants essential in protecting oils against oxidation. Vitamin E consists of four tocopherol forms (α‐, β‐, γ‐, and δ‐tocopherol) with saturated side chains, and four tocotrienol forms (α‐, β‐, γ‐, and δ‐tocotrienol) with unsaturated side chains containing three double bonds (Diamante et al. 2021).

Tocotrienols exhibit a broader spectrum of biological activities than their tocopherol counterparts, including antioxidant, anticancer, hypoglycemic, hypolipidemic, and cardioprotective properties. In particular, γ‐tocopherol, δ‐tocopherol, and γ‐tocotrienol have been associated with anti‐inflammatory activity through inhibition of cyclooxygenase‐ and 5‐lipoxygenase‐mediated eicosanoid synthesis, as well as attenuation of pro‐inflammatory signaling cascades (Ahsan et al. 2014; Tang et al. 2015; Wojdyło and Oszmiański 2020). The thermal stability of these compounds is also noteworthy: Butinar et al. (2011) observed that γ‐tocotrienol levels in pumpkin seeds were unaffected by roasting, with concentrations maintained at 6.9 ± 0.2 μg/g, a finding consistent with the retention of tocols observed in roasted açaí seed oil under the conditions applied in the present study.

Similarly, tocochromanols in oilseeds have been shown to retain considerable stability under moderate thermal and storage stress, with degradation becoming pronounced mainly under prolonged or more severe conditions (Gawrysiak‐Witulska et al. 2011).

### 3.4 Fatty Acid Profile and Acylglycerols Content

Table 3 presents the fatty acid profile of roasted açaí seed oil extract, in which myristic, oleic, linoleic, and palmitic acids were the predominant constituents. Saturated fatty acids account for 54.30% of the total, while unsaturated fatty acids constitute 42.98%. Da Silva et al. (2023) identified a fatty acid composition in non‐roasted açaí seed oil ( *Euterpe oleracea* Mart.) close to the values found here, with oleic acid (26.95%), linoleic acid (19.92%), myristic acid (18%), palmitic acid (16.61%), and lauric acid (9%) being prominent, with 49.27% saturated and 50.73% unsaturated fatty acids. The study by Melo et al. (2021) investigated the fatty acid composition of oil extracted from açaí ( *Euterpe oleracea* Mart.) seeds, reporting that approximately half of the lipid content consists of medium‐chain saturated fatty acids and the other half of long‐chain unsaturated fatty acids. The predominant fatty acids were myristic (22.86%), oleic (26.79%), and linoleic (22.52%), followed by palmitic acid (16.27%) and stearic acid (1.37%), with saturated fatty acids (SFA) totaling 49.24% and unsaturated fatty acids (UFA) totaling 50.76%. Similar results were found by Yuyama et al. (2011) for açaí from the species *Euterpe precatoria* Mart.

**TABLE 3:** Fatty acids profile and acylglycerol content of açaí seed oil extract.

Fatty acid	% w/w
Lauric (C12:0)	10.30
Myristic (C14:0)	25.97
Palmitic (C16:0)	16.71
Stearic (C18:0)	0.67
Oleic (C18:1, n‐9)	20.70
Linoleic (C18:2, n‐6)	21.41
Linolenic (C18:3, n‐3)	0.33
Arachidic (C20:0)	0.65
Erucic (C22:1, n‐9)	0.54
Others	2.72
MAG	0.79
DAG	5.89
TAG	93.32

TAG_Species_	Relative mole %
LiLiL	0.97
LiLiM	2.14
LiLiP	1.23
LOL	1.99
LOM	8.82
MOM	14.85
MOP	11.55
POP	4.08
MOA	0.82
LLiL	1.94
LLiM	8.59
MLiM	14.42
MLiP	12.05
PLiP	5.86
MOLi	4.32
PLiO	2.49
OOP	1.86

*Note:* Fatty acid values expressed as area percentage (% w/w). TAG species expressed as relative mol%; standard deviation does not apply to TAG values as they were estimated by combinatorial analysis.

Abbreviations: DAG, diacylglycerol; MAG, monoacylglycerol; TAG, triacylglycerol.

Myristic acid is the most abundant fatty acid identified in roasted açaí seed oil, a finding with both nutritional and industrial implications. On one hand, high intakes of myristic acid have been associated with elevated plasma LDL cholesterol and increased risk of cardiovascular disease and hepatic steatosis (Speziali et al. 2018). On the other hand, this fatty acid is not without physiological function: it serves as a substrate for protein myristoylation, a co‐translational modification that anchors signal transduction proteins to cell membranes and regulates enzyme activity (Rioux et al. 2011). In the food industry, myristic acid is used as a flavoring additive and antifoaming agent. Its concentration in roasted açaí seed oil (approximately 26%) is comparable to that found in coconut oil (approximately 21%; Kim et al. 2010), and considerably higher than in common vegetable oils such as sunflower, soybean, or canola, where it rarely exceeds 0.1%. The distinct fatty acid profile of Amazonian seed oils like this one represents a relevant opportunity for diversification in food, pharmaceutical, and cosmetic applications, particularly given that the seeds are generated on a large scale as agro‐industrial waste.

From a lipid stability perspective, the relatively high content of linoleic acid (18:2), a doubly unsaturated fatty acid highly susceptible to oxidation, raises important considerations regarding oil quality after roasting. Linoleic acid is known to undergo rapid autoxidation via free radical chain reactions at elevated temperatures, generating hydroperoxides and secondary oxidation products such as hexanal and malondialdehyde (Choe and Min 2006). Roasting at 140°C for 60 min constitutes a moderate thermal stress that may initiate oxidation, particularly in the presence of trace metals such as copper (as evidenced by the mineral profile of the seeds). Although oxidation indices (e.g., peroxide value, anisidine value, or conjugated dienes) were not determined in the present study, representing a recognized limitation, the low lipid content of the seeds (1.8%) likely limits the extent of bulk oxidation. Future studies should include measurement of primary and secondary oxidation markers to establish the oxidative stability of roasted açaí seed oil.

The acylglycerol profile of açaí seed oil, with TAG dominance (93.32%) and minor MAG (0.79%) and DAG (5.89%) fractions, suggests limited hydrolytic activity under the applied roasting and extraction conditions. The low MAG content indicates that extensive lipase‐mediated lipolysis did not occur, which is consistent with thermal inactivation of endogenous lipases during roasting at 140°C (Mazaheri et al. 2019; Shehu et al. 2019). The presence of DAG at 5.89% may reflect partial hydrolysis occurring either during seed storage, roasting, or hexane extraction at 50°C. Analysis of the TAG species revealed a diversity of compounds, each representing a specific proportion of the total composition: LiLiL (0.97%), LiLiM (2.14%), LiLiP (1.23%), LOL (1.99%), LOM (8.82%), MOM (14.85%), MOP (11.55%), POP (4.08%), MOA (0.82%), LLiL (1.94%), LLiM (8.59%), MLiM (14.42%), MLiP (12.05%), PLiP (5.86%), MOLi (4.32%), PLiO (2.49%) and OOP (1.86%).

Silva et al. (2019) conducted a study evaluating the TAG compositions of açaí ( *Euterpe oleracea* Mart.) pulp oil under three different extraction conditions. The main TAGs predicted, representing more than 85% of the total composition, included OOO, POO, OLiO, PLiO, and POP, with contents ranging from 25.26% to 27.97%, 26.16% to 28.44%, 12.34% to 15.15%, 8.37% to 10.45%, and 8.95% to 9.68%, respectively. In addition, Pereira et al. (2019) found TAGs such as OLiLi (2.8%), PLiP (1.3%), OLiO (13.6%), PLiO (7.1%), OOO (37%), POP (5.5%), and POO (24.5%) in bacaba (*Oenocarpus bacaba*) oil samples. Contextualizing within related *Euterpe* species, Silva et al. (2017) reported that jussara oil ( *E. edulis* Mart.) presented a TAG content of approximately 93.9%, with DAGs and MAGs + free fatty acids accounting for smaller fractions. Notably, the solvent used for extraction influenced the relative abundance of TAG classes; ethanol‐extracted oil yielded lower TAG purity but higher DAG content compared to ether extraction. A total of 14 TAG molecular species were characterized, with StOL and POL together comprising nearly half the total TAG pool in both solvent conditions. The much greater abundance of LLL in ethanol‐extracted oil was attributed to the higher polarity of ethanol selectively extracting more polar triacylglycerols. Across both extraction conditions, TAG profiles were qualitatively similar, with differences confined to the relative proportions of minor species (Silva et al. 2017). This profile differs substantially from that of roasted açaí seed oil, which is dominated by medium‐chain saturated TAGs (MOM, MLiM) rather than long‐chain oleic acid‐rich TAGs (OOO, POO), reflecting the distinct fatty acid composition between the two species.

### 3.5 Melting and Crystallization Profile

**FIGURE 3:**
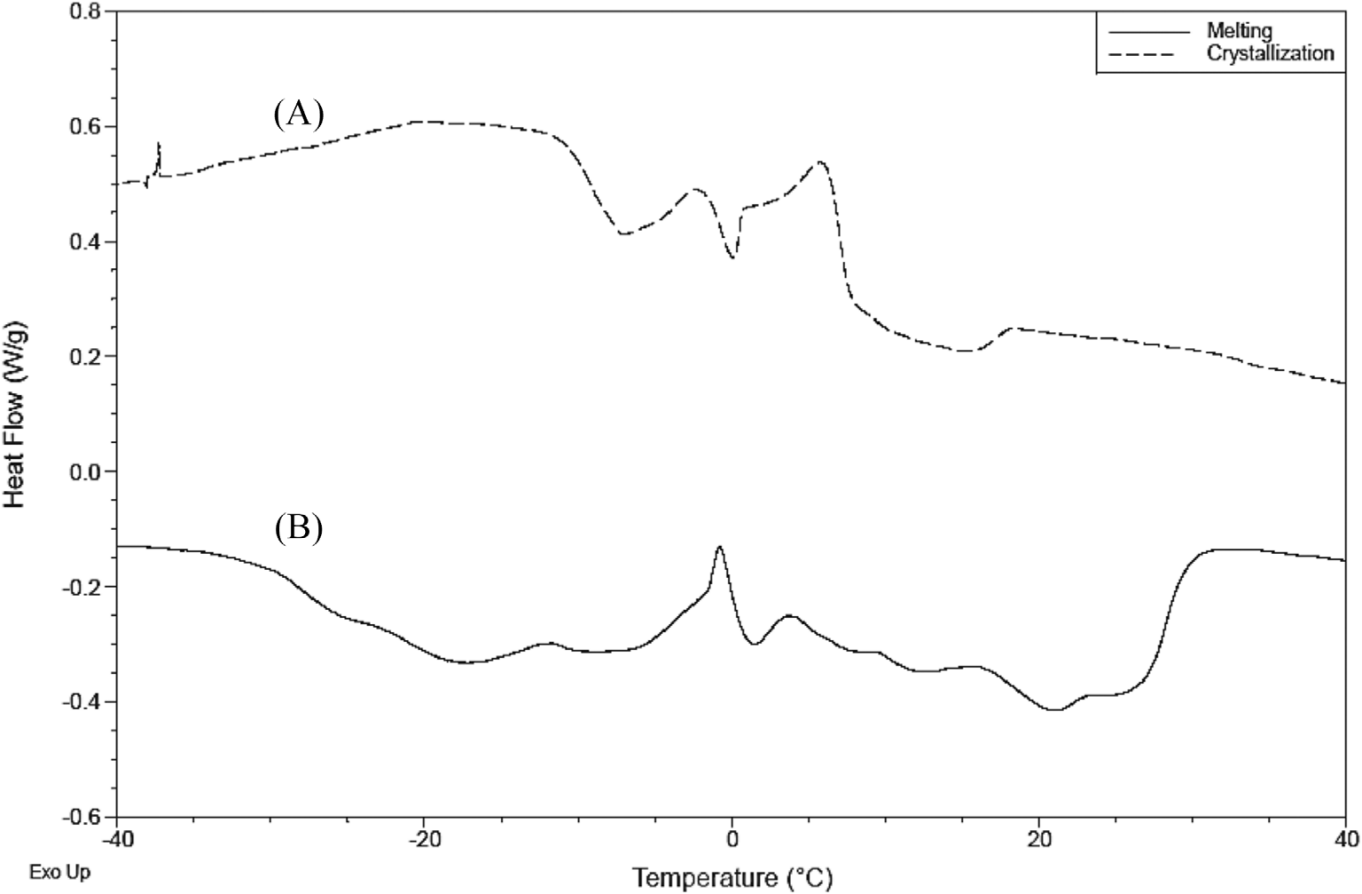
Crystallization (A) and melting curves (B) of pure fats and oil from roasted açaí seeds.

The thermograms showing the fusion and crystallization profiles of the roasted açaí seed oil extract are presented in Figure 3. Both curves exhibited multiple overlapping peaks across a wide temperature range, reflecting the complex and heterogeneous TAG composition of this oil. The coexistence of short‐to‐medium chain saturated fatty acids (lauric C12:0, myristic C14:0, palmitic C16:0) with long‐chain monounsaturated and polyunsaturated acids (oleic C18:1, linoleic C18:2), all individually exceeding 10% of the lipid profile, gives rise to a wide spectrum of TAG molecular species with distinct crystallization and melting temperatures. Complex multi‐peak DSC profiles are a hallmark of Amazonian seed oils with mixed fatty acid compositions, as also reported by Pereira et al. (2019) for Brazil nut oil, patawa oil, murumuru fat, tucuma kernel fat, pracaxi oil, and bacuri fat.

During cooling, the crystallization curve showed a series of exothermic events spanning from approximately 30°C to −40°C. Low‐intensity signals at higher temperatures (around 20°C–30°C) are attributed to the crystallization of di‐saturated TAGs containing palmitic acid (C16:0). More intense peaks near 0°C correspond to di‐saturated TAGs built from medium‐chain fatty acids such as myristic (C14:0) and lauric (C12:0). A broad, high‐intensity peak between −10°C and −40°C is consistent with TAGs containing medium‐chain saturated acids combined with linoleic acid (C18:2), whose unsaturation significantly depresses the crystallization temperature. The melting thermogram mirrored this complexity, with two broad transition regions: one spanning −40 to 0°C and another from 0 to approximately 30°C, attributed to TAGs rich in medium‐chain/polyunsaturated species and palmitic acid‐containing TAGs, respectively. Complete liquefaction near 30°C aligns with values reported for other Amazonian palm oils (Pereira et al. 2019). This thermal behavior, with a gradual solid‐to‐liquid transition spanning refrigeration to near‐ambient temperature, suggests practical utility in fat blend formulation and frozen product applications, where controlled melting is technologically desirable.

## 4 Conclusions

This study provides original physicochemical and compositional data on roasted açaí seeds powder ( *Euterpe oleracea* Mart.), a commercially available agro‐industrial by‐product that remains poorly characterized in the scientific literature, as well as aqueous and oil extracts obtained by them. The roasted powder showed nutritional relevance, with high dietary fiber and notable mineral concentrations. Elevated phenolic content was observed in both aqueous and oil extracts. Antioxidant capacity, assessed through complementary DPPH and FRAP assays, was substantial, and the tocol profile of the oil extracts dominated by α‐tocotrienol suggests that roasting enhances the extractability of lipophilic antioxidants from the seed matrix. The fatty acid and acylglycerol composition of the oil extracts, together with the complex thermal behavior observed by DSC, supports the potential of this product for food technology applications. Nevertheless, all antioxidant evaluations were performed in vitro, and the study was conducted on a commercially roasted batch under fixed conditions, which limits extrapolation to other processing scenarios. The absence of oxidative stability data is acknowledged as a recognized limitation. Future investigations should address variable roasting parameters, storage stability, additional antioxidant assays, seeds from distinct geographic origins and species, and bioaccessibility and bioavailability studies to fully establish the functional food potential of roasted açaí seed derivatives.

## Author Contributions

**Renata da Silva Magalhães:** investigation, methodology, writing – original draft, validation. **Gabriel Sthefano Lourenço Pereira:** formal analysis, writing – review and editing. **Sara Fraga:** formal analysis, writing – review and editing. **Patrícia Tonon de Souza:** formal analysis, investigation, methodology, writing – review and editing. **Dhayna Oliveira Sobral:** formal analysis, writing – review and editing. **Marcelo Antônio Morgano:** methodology, formal analysis, writing – review and editing. **Antonio José de Almeida Meirelles:** resources, visualization, writing – review and editing. **Guilherme José Maximo:** visualization, writing – review and editing. **Eduardo Augusto Caldas Batista:** resources, supervision, writing – review and editing, conceptualization. **Klicia Araujo Sampaio:** supervision, resources, writing – review and editing, conceptualization, funding acquisition, project administration.

## Funding

This work was supported by Fundação de Amparo à Pesquisa do Estado de São Paulo, 2014/21252‐0; 2017/12015‐3; 2022/10368‐4; 2014/50951‐4; 2024/03702‐0. Fundação de Amparo à Pesquisa do Estado do Amazonas, 01.02.016301.001624/2021‐10. Financiadora de Estudos e Projetos, 1310/24. Conselho Nacional de Desenvolvimento Científico e Tecnológico, 405813/2022‐8, 311994/2021‐0, 409532/2023‐1; 303622/2025‐3, 304152/2024‐2, 306298/2024‐4. Programa de Incentivo a Novos Docentes da UNICAMP, 519.287 2475/23; 519.287 2598/23. Coordenação de Aperfeiçoamento de Pessoal de Nível Superior, 001.

## Conflicts of Interest

The authors declare no conflicts of interest.

## Data Availability

The data that support the findings of this study are available on request from the corresponding author. The data are not publicly available due to privacy or ethical restrictions.
